# Chest CT in pediatrics: indications, findings, and use recommendations

**DOI:** 10.36416/1806-3756/e20260082

**Published:** 2026-03-05

**Authors:** Laura Zaffari Leal, Júlia Mundstock Noethen, Antônio Gomes Boabaid de Barros, Leonardo Araújo Pinto

**Affiliations:** 1. Grupo de Pesquisa em Epidemiologia e Genética das Doenças Respiratórias da Infância, Escola de Medicina, Pontifícia Universidade Católica do Rio Grande do Sul - PUCRS - Porto Alegre (RS) Brasil.; 2. Programa de Pós-Graduação em Medicina - Pediatria, Escola de Medicina, Pontifícia Universidade Católica do Rio Grande do Sul - PUCRS - Porto Alegre (RS) Brasil.

## INTRODUCTION

Respiratory diseases constitute one of the main causes of morbidity and mortality in pediatric patients. In this context, imaging methods are fundamental in the diagnosis and follow-up of these pathologies. Among routine clinical imaging methods for pulmonary evaluation, chest CT has the highest spatial resolution, allowing the precise identification of small structures in close proximity to each other. Therefore, chest CT is essential for identifying lung alterations such as bronchiectasis, peribronchial thickening, areas of air trapping, and congenital malformations.^1^ In this review, we summarize the main clinical indications and corresponding technical CT characteristics.

Despite its high diagnostic accuracy, chest CT presents some limitations when used in children and adolescents: exposure to ionizing radiation represents a concern for this population, because pediatric patients exhibit greater tissue radiosensitivity and a longer life expectancy, conditions that increase the cumulative risk of long-term stochastic effects, such as the development of neoplasms at another stage of life.^1^ In view of this, the indication for chest CT in such patients must follow a series of protection principles, primarily the as-low-as-reasonably-achievable concept, which advocates for the use of the lowest possible radiation dose without causing alterations in the image that compromise the diagnosis. In this context, other relevant aspects include the appropriate choice of acquisition protocols; the judicious use of iodinated contrast medium, which should be reserved for situations where there is a need for better vascular, mediastinal, or tissue characterization; and consideration of the need for sedation, especially in infants and young children. These factors must be carefully weighed according to the clinical indication.^1^ ^-^ ^3^

Indications, imaging findings, and radiation protocols in pediatric chest CT

## ACUTE CONDITIONS

### Infectious processes

In pediatric patients with infectious pulmonary conditions, the primary indication for chest CT is the investigation of complications not adequately identified by conventional radiography. In immunocompetent patients with complicated pneumonia, the main CT findings include areas of low attenuation within a consolidated parenchyma, suggestive of lung abscess or cavitary necrosis, as well as the presence of air in the pleural space, indicating possible bronchopleural fistula formation.

In immunocompromised patients, CT should be employed more frequently, given the high morbidity and mortality associated with respiratory infections in this group. In this population, pneumonia caused by uncommon etiologic agents, such as fungi, is more common and is often accompanied by the halo sign or the air crescent sign. A diffuse ground-glass pattern suggests viral etiologies or *Pneumocystis jirovecii* infection. In pediatric patients, pulmonary tuberculosis typically presents with cavitations (in adolescents), dense consolidations, and calcified lymphadenopathy.^4^ In these situations, CT protocols involving low radiation doses, which are sufficient for identifying infectious complications, are preferred. In addition, the use of intravenous contrast is reserved for selected cases, such as those of abscess or mediastinal involvement.^2^ ^,^ ^5^ Furthermore, chest CT is not indicated in non-severe, non-complicated acute bacterial pneumonia in pediatric patients.

## DIFFUSE AND CHRONIC LUNG DISEASES

### Interstitial lung diseases

Although chest X-ray is the first-line examination in the evaluation of interstitial lung diseases, the gold standard for characterizing the pattern of involvement and for guiding biopsies is HRCT. Although many findings, such as a ground-glass pattern, are nonspecific, HRCT contributes significantly to narrowing the diagnostic hypotheses when interpreted together with the clinical data.^6^ Furthermore, an HRCT scan assists in directing more invasive diagnostic procedures, such as bronchoalveolar lavage or lung biopsy, by allowing the identification of the most affected areas and the extent of pulmonary involvement. For pediatric patients with established chronic interstitial lung disease requiring serial assessment, the use of “limited HRCT” is recommended. That limited protocol significantly reduces radiation exposure while maintaining the diagnostic reliability essential for longitudinal follow-up.^4^

### Specific chronic pathologies

Chest CT plays a fundamental role in chronic pediatric lung diseases, being used not only for the initial diagnosis but also for monitoring disease progression and treatment response. In cystic fibrosis, notable findings include bronchiectasis with a predominance in the upper lobes, bronchial wall thickening, mucoid impaction, a “tree-in-bud” pattern, and mosaic attenuation. That last finding is also characteristic of bronchiolitis obliterans, which manifests as a diffuse pattern of mosaic attenuation together with parenchymal bands, often requiring expiratory slices to confirm air trapping. Similarly, bronchopulmonary dysplasia presents with coarse interstitial lines associated with ground-glass opacities and cystic changes. In cases of severe asthma categorized as difficult to control, CT may show airway remodeling, characterized by bronchial thickening, bronchiectasis, and indirect signs of small airway involvement.^4^ In this group of diseases, low-dose chest CT protocols are recommended, which allow adequate evaluation of structural pulmonary changes with a lower cumulative radiation dose. More recently, ultra-low-dose chest CT has been employed, using advanced reconstruction algorithms to further minimize radiation exposure. The ultra-low-dose protocol proves particularly suitable for screening examinations and for the follow-up of chronic lung diseases, maintaining sufficient diagnostic information for clinical monitoring with an additional dose reduction in comparison with conventional low-dose CT.^5^

[Table t1]

**Table 1 t1:** Summary of characteristic chest CT findings and techniques according to the clinical indications described in this article.

Clinical indication	Characteristic findings	Technique
Infectious diseases		
Complicated pneumonia	Parenchymal areas of low-attenuation (suggestive of abscess or necrosis); pleural air collection (indicating possible bronchopleural fistula)	Low-dose chest CT
Pulmonary tuberculosis	Cavitary lesions; dense consolidations; calcified lymphadenopathy	Low-dose chest CT
Diffuse and chronic lung diseases		
Interstitial lung diseases	Ground-glass opacities; reticular pattern; interstitial thickening; areas of fibrosis; distribution and extent of parenchymal involvement	HRCT of the chest
Cystic fibrosis	Upper lobe-predominant bronchiectasis; bronchial wall thickening; mucoid impaction; tree-in-bud pattern; mosaic attenuation	Low-dose or ultra-low-dose chest CT may be used for screening and follow-up
Bronchiolitis obliterans	Diffuse mosaic attenuation; parenchymal bands; air trapping (more evident on expiratory imaging)	
Congenital pulmonary malformations	Well-defined congenital parenchymal mass or cystic lesion; abnormal pulmonary architecture; systemic arterial supply on contrast-enhanced CT	Volumetric chest CT, adjusted to the lowest possible dose

## CONGENITAL PULMONARY MALFORMATIONS

In congenital pulmonary malformations, CT is the examination of choice because it provides essential anatomical detail for surgical planning or conservative management. In these cases, volumetric CT protocols are used, adjusted to the lowest possible dose. In asymptomatic newborns with a prenatal diagnosis, it is recommended that the examination be postponed until after the neonatal period to reduce initial radiation exposure. In pulmonary sequestration, Doppler ultrasound can be used for the initial evaluation, allowing CT to be delayed until a time closer to the surgical procedure. In this situation, contrast-enhanced chest CT is decisive in demonstrating the systemic origin of the feeder artery, generally from the aorta, as well as the associated venous drainage pattern.^4^

## CONCLUSION

The use of chest CT in the pediatric population encompasses a broad spectrum of clinical indications. Mastery of the appropriate indications, accurate recognition of imaging findings, and tailored technical approaches are essential to optimize diagnostic performance while minimizing radiation exposure. A thorough understanding of these aspects enables clinical management that is more precise and individualized.
